# Trans- and Within-Generational Developmental Plasticity May Benefit the Prey but Not Its Predator during Heat Waves

**DOI:** 10.3390/biology11081123

**Published:** 2022-07-27

**Authors:** Andreas Walzer, Gösta Nachman, Bernhard Spangl, Miroslava Stijak, Thomas Tscholl

**Affiliations:** 1 University of Natural Resources and Life Sciences, Vienna, Department of Crop Sciences, Institute of Plant Protection, Gregor-Mendel-Straße 33, 1180 Vienna, Austria; andreas.walzer@boku.ac.at (A.W.); miroslava.stijak@students.boku.ac.at (M.S.); 2 Department of Biology, University of Copenhagen, Universitetsparken 15, DK-2100 Copenhagen Ø, Denmark; gnachman@bio.ku.dk; 3 University of Natural Resources and Life Sciences, Vienna, Department of Landscape, Spatial and Infrastructure Sciences, Institute of Statistics, Peter-Jordan-Straße 82/I, 1190 Vienna, Austria; bernhard.spangl@boku.ac.at

**Keywords:** phytoseiidae, tetranychidae, biological control, climate change, developmental plasticity, heat stress, intergenerational plasticity, predator prey interactions

## Abstract

**Simple Summary:**

Heat waves can have fatal effects on arthropods such as insects and mites since their heat tolerance is often lower than the diurnal maximum temperatures during heat waves. Plastic modifications by the parents, however, can rapidly result in favorable adaptations in offspring traits. This question was investigated by using a prominent natural enemy/pest couple in biological control, the predatory mite *Phytoseiulus persimilis* and its prey, the spider mite *Tetranychus urticae*. We exposed both species separately to extreme or mild heat waves during their juvenile development, a vital phase of arthropod life, for two generations and assessed various fitness-relevant parameters of the offspring generation. Under extreme heat waves, adult body sizes of predator and prey males and prey females were insensitive, when they derived from parents also reared under extreme heat waves. Irrespective of their origin, offspring reached earlier adulthood under extreme heat waves. In general, prey benefitted more from parental modifications compared to the predator. However, further investigations are needed to verify whether these changes affect the interactions between the predators and their prey to an extent that it may jeopardize biological control during extreme heat waves.

**Abstract:**

Theoretically, parents can adjust vital offspring traits to the irregular and rapid occurrence of heat waves via developmental plasticity. However, the direction and strength of such trait modifications are often species-specific. Here, we investigated within-generational plasticity (WGP) and trans-generational plasticity (TGP) effects induced by heat waves during the offspring development of the predator *Phytoseiulus persimilis* and its herbivorous prey, the spider mite *Tetranychus urticae,* to assess plastic developmental modifications. Single offspring individuals with different parental thermal origin (reared under mild or extreme heat waves) of both species were exposed to mild or extreme heat waves until adulthood, and food consumption, age and size at maturity were recorded. The offspring traits were influenced by within-generational plasticity (WGP), trans-generational plasticity (TGP), non-plastic trans-generational effects (TGE) and/or their interactions. When exposed to extreme heat waves, both species speeded up development (exclusively WGP), consumed more (due to the fact of WGP but also to TGP in prey females and to non-plastic TGE in predator males), and predator females got smaller (non-plastic TGE and WGP), whereas prey males and females were equally sized irrespective of their origin, because TGE, WGP and TGP acted in opposite directions. The body sizes of predator males were insensitive to parental and offspring heat wave conditions. Species comparisons indicated stronger reductions in the developmental time and reduced female predator-prey body size ratios in favor of the prey under extreme heat waves. Further investigations are needed to evaluate, whether trait modifications result in lowered suppression success of the predator on its prey under heat waves or not.

## 1. Introduction

Extreme high temperature events, such as heat waves, are becoming more common because of ongoing climate warming [1]. Additionally, the intensity and duration of heat waves are expected to increase over the next decades [2], which pose serious challenges for ectothermic species. Unlike the slow increase in the mean temperatures, daily temperature maxima occur within hours during heat waves and often exceed the critical maximum temperatures for their juvenile development, reproduction and survival [3]. Accordingly, fast responses of the exposed individuals are needed to cope with heat waves, making plastic effects without changing the genetic code more likely than slow genetic adaptations [4,5]. Such within-generational phenotypic changes [within-generational plasticity (WGP)] of fitness-relevant traits, induced by heat waves, are well documented in several ectothermic species [6,7,8,9,10]. However, the climatic conditions experienced by parents are increasingly recognized to also influence traits of the next generation via non-genetic modifications, termed trans-generational plasticity (TGP) [11,12]. The occurrence of trans-generational effects should be promoted by high environmental heterogeneity, the precise identification of cues indicating environmental changes, and the, at least partly, matching of parental and offspring environment [13,14]. Under these aspects, heat waves could be ideal drivers of TGP effects in ectotherms with fast generation sequences. First, the irregular appearance of heat waves creates a temporally patchy environment, which should favor plasticity within, but also across generations [15,16]. Second, the daily temperature peaks are reliable cues indicating heat waves, and ectotherms usually have highly sensitive thermo-receptors to detect temperature changes [17,18,19]. Additionally, ectotherms have also confronted with heat waves in the past, such that they should detect and identify them correctly [20]. Third, parental climatic conditions are more predictive of offspring climatic conditions in species with short generation times, because fast juvenile development increases the temporal correlation of heat wave experience between parents and their offspring [21].

Early life stages are usually highly sensitive to heat waves, indicating that TGP effects on juvenile development are expected to have an advantage over WGP effects, because the latter response requires a mechanism for reliable detection of heat stress. Early life stages may lack suitable sensory organs causing a time lag of plastic responses. In contrast, trans-generational developmental plasticity should lead to faster plastic responses, improving the survival probabilities of the heat-stressed offspring early in life [15]. Consequently, TGP effects on juvenile development may allow ectothermic species to keep pace with rapid environmental changes such as heat waves [22]. Nonetheless, evidence for TGP effects induced by heat waves are still rare in relation to juvenile development of ectotherms [4,23,24,25,26], which could cause shifts in age and size at maturity. Such species-specific plastic modifications may play a critical role in predator-prey interactions, when the counterparts have different or even opposite plastic capacities to heat waves [27]. Reaching earlier adulthood by the predator, but not prey, means more encounters of juvenile prey with adult predators, which are normally the most aggressive and voracious predator stages. Contrary, faster prey development can reduce the time slot for predation on early prey life stages. Opposing thermal plasticity effects on predator and prey in relation to size at maturity can change predator-prey body size ratios [28], a pivotal parameter for predation success [29].

We studied potential trans-generational heat wave effects on the juvenile development of the predatory mite *Phytoseiulus persimilis* Athias-Henriot (Acari: *Phytoseiidae*) and its preferred prey, the spider mite *Tetranychus urticae* Koch (Acari: *Tetranychidae*). The two species are prominent counterparts in biological control [30]. Both species pass through three mobile stages (i.e., larva, protonymph and deutonymph), until they reach adulthood in a few days under extreme heat waves [10]. These plant-inhabiting mites are confronted with a heterogeneous environment in relation to biotic and abiotic factors. As a consequence, WGP and TGP effects on their juvenile development are frequently induced by food limitation [31,32,33], predation risk [34,35,36,37], temperature [38,39,40] and humidity [41,42,43]. These findings indicate the high capacity of both species for plastic responses to cope with rapid environmental changes. Not surprisingly, within-generational developmental effects triggered by heat waves resulted in shifts in the age and size at maturity in both species. However, prey should benefit more from the plastic modifications reflected in higher developmental gain rates and a lowered female predator-prey body size ratio [10]. So far, there is no evidence for trans-generational effects on juvenile development as a response to heat waves in either species. Such thermal trans-generational effects can lead to changes of offspring developmental modifications, which can differ in their magnitude and direction compared to WGP effects experienced by the parental generation. For example, the females of another predatory mite, *Amblydromalus limonicus* (Acari: *Phytoseiidae*), reached later adulthood and with smaller sizes when confronted with heat waves. In contrast, their heat wave-exposed daughters speeded up development and reached larger body sizes because of trans-generational effects [4]. Thus, parental trait modifications in the offspring generation may outweigh costs of the parental generation via fitness increase of their descendants. 

Based on these considerations, we hypothesized that (1) dependent on the parental heat wave exposure (mild or extreme), both the predator and prey offspring generation react to heat waves with a combination of trans-generational and within-generational responses, thereby changing life history traits; (2) these plastic modifications may in general provide benefits for offspring; (3) during extreme heat waves the prey *T. urticae* profits more from thermal phenotypic plasticity than the predator *P. persimilis*.

## 2. Materials and Methods

### 2.1. Mite Origin and Rearing

Predatory and spider mites from a commercial producer of natural enemies (Biohelp, Vienna, Austria) were used to set up laboratory colonies. Spider mites were reared on bean plants (*Phaseolus vulgaris* L.) and predatory mites were reared on plastic tiles supplied with bean leaves infested by spider mites. Both species were raised in the lab for three years, which corresponds to approximately 120 predator- and 80 prey generations. The climatic conditions were the following: spider mites = 25 ± 2 °C, 60 ± 15% relative humidity (RH), 16 h:8 h; predatory mites = 25 ± 1 °C, 60 ± 10% RH, 16 h:8 h (for rearing details, see [10]).

### 2.2. Heat Wave Conditions

In Austria, the spider mite predator *P. persimilis* is mainly used in the eastern parts, where the main areas for growing vegetables and soft fruits are located. Thus, an analysis of the heat waves from 2011 to 2020 in this region was conducted [10] in order to identify natural heat wave characteristics. Based on these findings, we defined two heat wave scenarios: the mild (M), characterized by *T*_mean_ = 22.6 °C, *T*_max_ = 32.0 °C, *T*_min_ = 16 °C, RH_mean_ = 67.9%, RH_max_ = 85.0%, RH_min_ = 50.0%, and the extreme (E), characterized by *T*_mean_ = 28.6 °C, *T*_max_ = 38.0 °C, *T*_min_ = 22.0 °C, RH_mean_ = 60.0%, RH_max_ = 75.0%, RH_min_ = 50.0%. Light:dark conditions in both scenarios were 16 h:8 h. The two scenarios were chosen to represent the conditions prevailing during present and prospective heat waves in eastern Austria, assuming continued global warming (for more details see [10]). The exact temperature and humidity values for mild and extreme heat waves are depicted in Figure S1 (supplementary material).

### 2.3. Experimental Setup

First, young, gravid females from the lab colonies were placed in cohorts of 20 predator or prey individuals on separate large, detached bean leaves (96 to 140 cm^2^). Predatory mites were provided with ample prey eggs. The females were allowed to deposit eggs at constant 25 °C during 6 h and then removed. These eggs were then exposed to extreme (E) or mild (M) heat wave conditions until reaching adulthood. The young, gravid females of the parental generation (F0) were then placed on detached bean leaves for 6 h and allowed to lay eggs, which were the study individuals of the offspring generation (F1).

**Figure 1:**
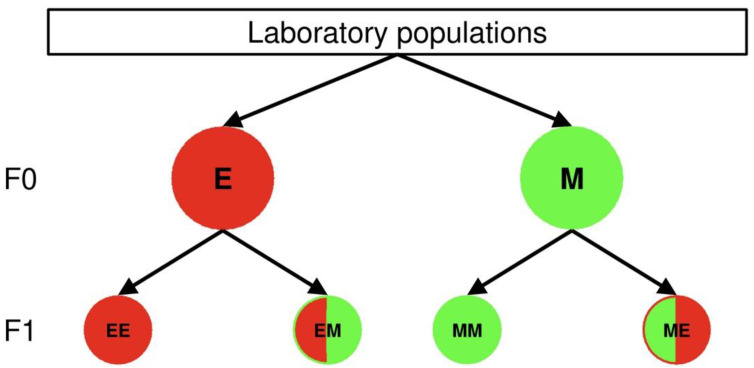
Rearing conditions of the parental (F0) and offspring generation (F1) during juvenile development. Parental individuals derived from females of the lab populations and raised under extreme (E, red F0 cycle) or mild heat waves (M, green F0 cycle) until adulthood. The parental females were then allowed to deposit the offspring eggs, which were either exposed to extreme or mild heat wave conditions for their whole juvenile development. The first and second upper case letters in the F1 cycles are indicating the parental and offspring heat wave conditions during juvenile development, respectively. The colors of the F1 cycles indicate, whether parental heat wave conditions are matching offspring conditions (uniformly colored cycles) or not (bicolored cycles). In the latter case the colors in the left and right semi-cycles indicate the parental and offspring heat wave conditions, respectively. The following combinations of the parental and offspring heat wave conditions were evaluated: (1) parents and offspring developed under extreme heat waves (EE, uniformly red colored), (2) parents developed under extreme heat waves, offspring under mild heat waves (EM, red and green colored), (3) parents and offspring developed under mild heat waves (MM, uniformly green colored), (4) parents developed under mild heat waves, offspring under extreme heat waves (ME, green and red colored).

These predator or prey eggs were put singly on small bean leaves (2.5 × 2.5 cm), which were placed on a water-saturated foam cube inside a plastic box. Predators were provided with 40 spider mite eggs shortly before the larval hatch, because the protonymphal stage of the predator is the first feeding stage. The amount of prey is sufficient for the whole juvenile developmental period of *P. persimilis* [10]. A glue (fruit tree grease, Vitax) margined the arena and prevented the mites from escaping, while a folded plastic piece served as refuge on the experimental units with *P. persimilis*. Depending on parental and offspring thermal environments, the four heat wave combinations of the offspring predator and prey eggs were labelled MM, ME, EM and EE, where the first letter refers to the thermal conditions to which the parents were exposed and the second to the offspring conditions experienced during juvenile development (Figure 1). The experimental units with the offspring eggs were placed in the programmable incubator MLR-352H-HE (temperature variation: ±0.5 °C, humidity variation: ±5% RH) during their entire juvenile period.

### 2.4. Evaluated Traits

Before the start of the experiments, the volume of the offspring eggs was assessed by photographing them with the microscope system Leica DMS 1000 and then measuring their diameter (for spheroid *T. urticae* eggs) or length and width (for prolate ellipsoid *P. persimilis* eggs) with the software Leica Application Suite X (LAS X 3.7.4.23463). These measurements were used to calculate the egg volume for *T. urticae* as $V_{\mathrm{TU}}=\frac{4}{3}\cdot \pi \cdot {\left(\frac{d}{2}\right)}^{3}$ and for *P. persimilis* as $V_{\mathrm{PP}}=\frac{4}{3}\cdot \pi \cdot \frac{l}{2}\cdot {\left(\frac{w}{2}\right)}^{2},$ where *d* denotes the diameter of a spider mite egg, while *l* and *w* denote the length and width of a predatory mite egg. The sex of an egg was determined after the larvae hatched and reached adulthood. 

The state (i.e., dead or alive), developmental progress of predator and prey, and the food consumption of the predator (number of eaten spider mite eggs) were observed in 8 and 16 h intervals per day. The developmental rate was calculated as the inverse to the time required to develop from egg to adulthood. Total food consumption by *T. urticae* was assessed after adult enclosure by measuring the area of feeding scars by means of a transparent mm^2^ screen. Sex and adult body size were determined after placing adult mites in a drop of Hoyer’s medium. Body size was measured as the perimeter of the dorsal shield for *P. persimilis* and the idiosoma for *T. urticae*, using body setae as orientation (for details, see [10]). 

### 2.5. Statistical Analyses

Statistical analyses were performed with SAS on Demand for Academics [SAS Inst. 2021, Cary (USA)]. First, logistic regression (PROC LOGISTIC) was used to analyze whether survival probability and sex ratio (proportion of females) of offspring were affected by the heat wave conditions experienced by the parents and their offspring, as well as by the interaction between the two factors, assuming binomially distributed data and a logit link function. Each species was analyzed separately. The GOF option was used to assess the goodness-of-fit based on the Hosmer-Lemeshow test [44]. Agreement with the logistic model was accepted for *p* > 0.05.

Second, the parental heat wave effects on eggs resulting in viable offspring were analyzed for each species by modeling egg volume by means of a two-way ANOVA (PROC GLM) with the parental heat wave conditions and offspring sex, as well as their interaction as explanatory variables. 

**Figure 2:**
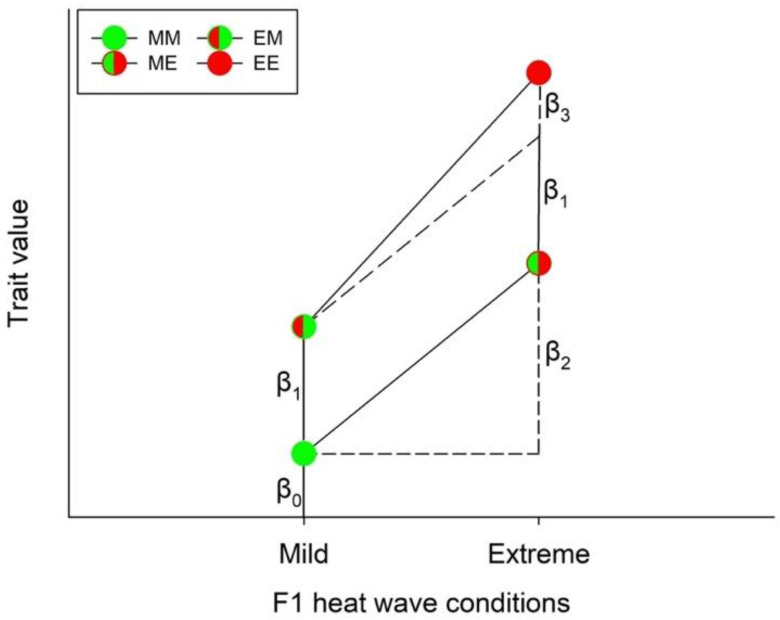
Schematic representation of the model used to analyze for potential plasticity types induced by heat waves (or other environmental stressors). The circles illustrate the different heat wave combinations. Each circle was divided into two halves: the left half marks whether the F0 generation was exposed to mild (green) or extreme (red) heat wave conditions, while the right half marks whether the offspring (F1) was exposed to mild (green) or extreme (red) heat wave conditions. *β*_0_ is the model’s intercept (i.e., the expected trait value (*y*) when both F0 and F1 were exposed to mild heat waves, corresponding to F0 = F1 = 0 in Eqn 1). If only *β*_1_ was significantly different from 0, it indicates a non-plastic trans-generational effect (TGE). A significant *β*_2_ indicates a within-generational plastic response (WGP), while a significant *β*_3_ indicates a trans-generational plastic response (TGP). Since the parameters can take both positive and negative values, the combined response to treatments will be largest if the parameters have the same sign and smallest if they differ with respect to sign.

Third, each species and sex were analyzed separately with respect to how the offspring produced by parents exposed to either mild or extreme heat waves responded to thermal conditions, using the model,

(1) $$y=\beta_{0}+\beta_{1}F0+\beta_{2}F1+\beta_{3}F0*F1+\varepsilon$$

where *F*0 and *F*1 are dummy variables expressing the thermal conditions experienced by the parents and their offspring, respectively. Thus, *F*0 and *F*1 can be either 0 or 1, depending on whether conditions were mild (M) or extreme (E). The *β*s represent the model’s parameters (Figure 2). The significance of the individual parameters was tested by means of *t*-tests. A significant *β*_1_ (i.e., if *p* < 0.05) is interpreted as heat wave conditions experienced by the parents influenced their offspring’s performance (indicating non-plastic trans-generational effects—TGE), while a significant *β*_2_ is interpreted as the offspring responded to the environment in which they grew up (indicating WGP). Finally, if the interaction term (*β*_3_) was significantly different from 0, this indicates the presence of TGP (Figure 2). *F*0wass regarded as a blocking factor reflecting that the parents constituted a random sample of parents with different genetic backgrounds but exposed to the same environment. Thus, *F*0 is treated as a random factor while *F*1 is a fixed factor. The analysis was carried out by means of PROC GLM. The assumptions for normality and variance homogeneity were assessed visually via quantile-quantile (QQ) plots. Pairwise post hoc comparisons between treatments were carried out by means of Tukey’s HSD test using the LSMEANS statement. 

Fourth, the ratios between adult predators and prey of both sexes with respect to body size and developmental time were analyzed according to Eqn 2.7.16 in Colquhoun [45] using PROC MEANS to calculate variances and the 95% confidence limits associated with different treatment combinations. The difference between two treatments was tested by means of *t*-tests and multiple pairwise tests were conducted by means of Bonferroni adjustments. 

Fifth, we calculated the change in the average developmental rates for both species (pooled over sex), when heat wave conditions changed from mild to extreme in order to compare how the two species responded to increasing temperatures and determine out whether their responses depended on how their parents were treated. The 95% confidence limits for the ratio between two developmental rates were obtained in the same way as above. 

Trait values are presented in the text as averages followed by the 95% confidence limits in square brackets, and as dots and vertical lines in the figures.

## 3. Results

### 3.1. Heat Wave Effects on Survival and Sex Ratios

The survival rates of both the predator (MM: 0.882 [0.788–0.937], ME: 0.946 [0.864–0.980], EM: 0.842 [0.757–0.901] and EE: 0.890 [0.802–0.942]) and the prey (MM: 0.899 [0.810–0.949], ME: 0.934 [0.861–0.970], EM: 0.850 [0.754–0.913] and EE: 0.930 [0.853–0.968]) were high and not affected by parental or offspring heat wave conditions and their interaction (Table 1). Additionally, parental and offspring heat wave conditions and their interaction did not affect the sex ratios of the mites (Table 1). In detail, the predator sex-ratios (females:males) of offspring were: MM: 39:28 = 0.582 [0.462–0.694], ME: 45:23 = 0.662 [0.542–0.764], EM: 48:37 = 0.565 [0.458–0.666], EE: 48:25 = 0.658 [0.542–0.757] while the corresponding values for the prey were: MM: 35:36 = 0.493 [0.379–0.608], ME: 50:35 = 0.588 [0.481–0.688], EM: 40:28 = 0.588 [0.468–0.698], and EE: 41:39 = 0.513 [0.404–0.620].

### 3.2. Effects of Parental Heat Wave Conditions on Offspring Egg Sizes

**Figure 3:**
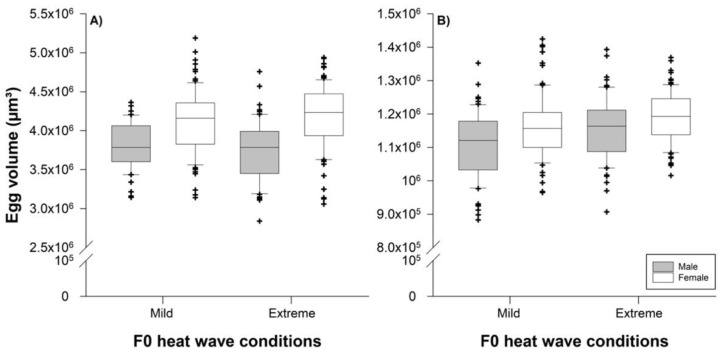
Effects of the parental (F0) heat wave conditions (reared under mild or extreme waves during juvenile development) on offspring male (grey boxes) and female (white boxes) egg volumes of *P. persimilis* (**A**) and *T. urticae* (**B**). The horizontal centerlines in each box represent the median, the box limits represent the interquartile (IQ) range from the 25th to 75th percentiles, the whiskers extend the IQ range to 1.5 times and outliers are depicted as symbols.

Predator egg sizes were significantly influenced by their sex (*F*_1,289_ *=* 64.90; *p* < 0.001), but not by parental heat wave conditions (*F*_1,289_ *=* 0.00; *p* = 0.9658) or the interaction between the two factors (*F*_1,289_ *=* 1.93; *p* = 0.1658). Female eggs were on average larger than male eggs (4.150 [4.089–4.210] × 10^6^ µm^3^ versus 3.765 [3.699–3.831] × 10^6^ µm^3^) (Figure 3A).

Prey egg sizes were affected by both their sex (*F*_1,300_ = 18.08; *p* < 0.001) and parental heat wave conditions (*F*_1,300_ = 11.72; *p* = 0.001), but not by their interaction (*F*_1,300_ = 0.71; *p* = 0.400). Female eggs were on average larger than male eggs (1.176 [1.162–1.189] × 10^6^ µm^3^ versus 1.158 [1.114–1.148] × 10^6^ µm^3^) and eggs were slightly larger when laid by females developing to adulthood under extreme heat wave conditions compared with mild conditions (1.173 [1.158–1.189] × 10^6^ µm^3^ versus 1.138 [1.123–1.153] × 10^6^ µm^3^) (Figure 3B).

### 3.3. Heat Wave Effects on Offspring Food Consumption, Age and Size at Maturity

**Figure 4:**
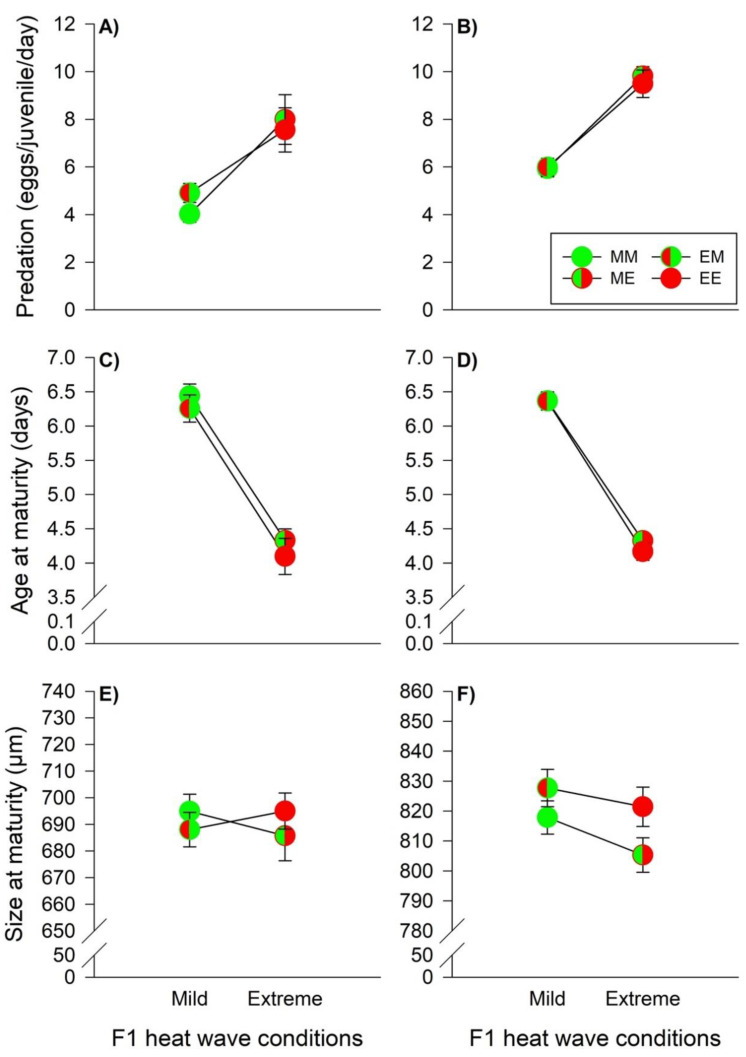
Heat wave effects during juvenile development on the predation rate, age at maturity, and size at maturity of males (**A**,**C**,**E**) and females (**B**,**D**,**F**) of the predator *P. persimilis*. The circles illustrate the different heat wave combinations. Each circle is divided into two halves: the left half marks whether the parents (F0) were exposed to mild (green) or extreme (red) heat wave conditions, while the right half marks whether the offspring (F1) were exposed to mild (green) or extreme (red) heat wave conditions. Offspring, originating from parents reared under mild (M) or extreme (E) heat waves, and offspring exposed to mild (M) or extreme (E) heat waves are labeled by the first and second upper case letters, respectively (MM = 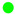, EM = 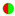, ME = 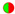, EE = 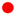). The lines connecting two circles refer to offspring experiencing the same heat wave condition as their parents. Vertical lines show the 95% confidence limits.

Food consumption of the juvenile predators was primarily influenced by the offspring heat wave conditions, while males also showed indications of TGE (Table 2). Male and female juveniles exhibited an increase in egg consumption, when they grew up under extreme heat wave conditions, indicating WGP effects (males, mild versus extreme heat waves: 4.53 [4.24–4.82] eggs/day versus 7.77 [7.07–8.46] eggs/day, females: 5.96 [5.71–6.22] eggs/day versus 9.65 [9.30–10.00] eggs/day) (Figure 4A,B). Along the same line, predator age at maturity of males and females was strongly influenced by offspring heat wave conditions (Table 2). Both male and female development were faster, when the predators grew up under extreme heat waves (males, mild versus extreme heat waves: 6.34 [6.20–6.47] days versus 4.21 [4.05–4.37] days, females: 6.37 [6.27–6.46] days versus 4.24 [4.16–4.32] days) (Figure 4C,D). Male predator size at maturity was not influenced by parental- and offspring heat wave conditions. The interaction term was significant indicating TGP effects, but since *β*_2_ and *β*_3_ had different signs, the pairwise tests (MM versus ME and ME versus EE) did not provide significant differences (Table 2, Figure 4E). Female body sizes were affected by parental- and offspring heat wave conditions, but not by their interaction (Table 2). Females originating from parents reared under extreme heat waves were larger, indicating non-plastic TGE (824.57 [820.05–829.09] μm versus 810.83 [806.57–815.09] μm). Additionally, WGP effects induced size reductions in females under extreme heat waves (813.36 [808.66–818.05] μm versus 823.29 [818.97–827.61] μm) (Figure 4F). 

**Figure 5:**
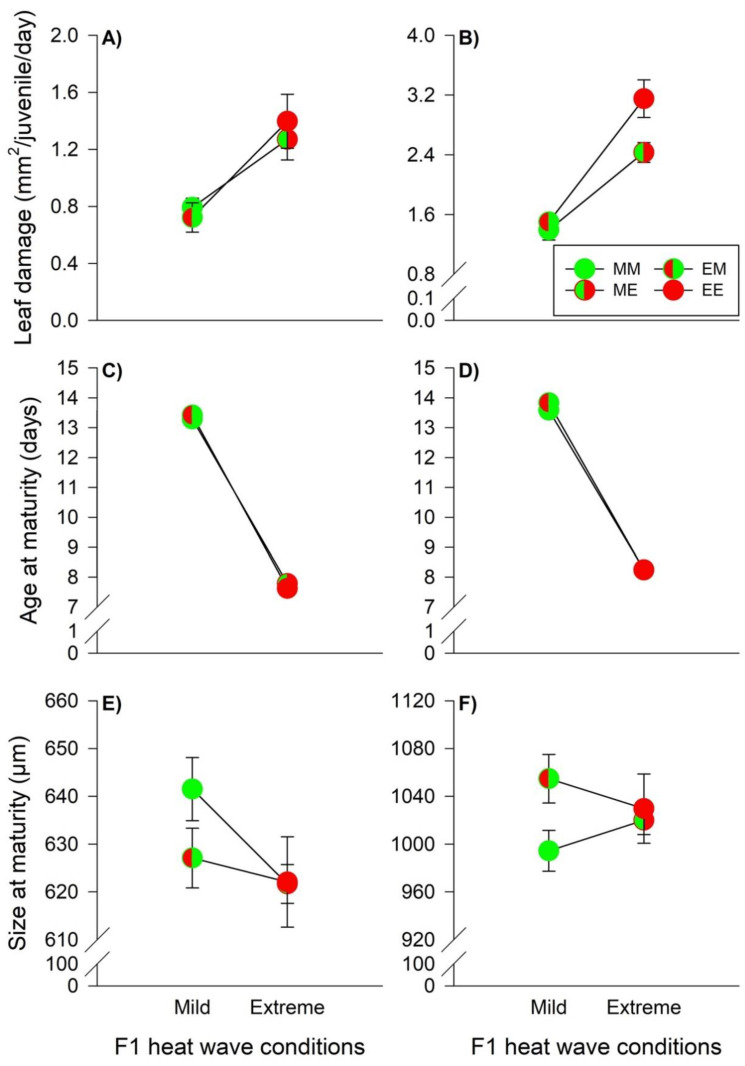
Heat wave effects during juvenile development on the leaf damage, age at maturity, and size at maturity of males (**A**,**C**,**E**) and females (**B**,**D**,**F**) of the prey *T. urticae*. The circles illustrate the different heat wave combinations. Each circle is divided into two halves: the left half marks whether the parents (F0) were exposed to mild (green) or extreme (red) heat wave conditions, while the right half marks whether the offspring (F1) were exposed to mild (green) or extreme (red) heat wave conditions. Offspring originating from parents reared under mild (M) or extreme (E) heat waves and offspring exposed to mild (M) or extreme (E) heat waves are labelled by the first and second upper case letters, respectively (MM = 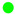, EM = 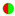, ME = 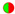, EE = 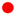). The lines connecting two circles refer to offspring experiencing the same heat wave condition as parents. Vertical lines show 95% confidence limits.

Food consumption by male prey was only affected by WGP effects, because extreme heat waves led to higher consumption rates irrespective of male origin (mild versus extreme heat waves: 0.76 [0.70–0.82] mm^2^ versus 1.34 [1.22–1.56] mm^2^) (Table 3, Figure 5A). Female food consumption was influenced by offspring heat wave conditions and the interaction between parental and offspring conditions (Table 3). WGP effects became manifest in higher consumption rates when females were exposed to extreme heat waves (MM versus ME: *t*_162_ = 8.917; EM versus EE: *t*_162_ = 14.08; both *p* < 0.0001). However, the offspring heat wave effects were also dependent on the parental heat wave conditions, because consumption rates were higher under extreme heat waves in females originating from parents reared under extreme heat waves (EE versus ME: *t*_162_ = 6.481; *p* < 0.0001), indicating also TGP effects (Figure 5B). Age at maturity of male and female prey was influenced by offspring heat wave conditions, while females were also slightly affected by TGE (Table 3). WGP effects became manifest in faster prey development under extreme heat wave conditions (males, mild versus extreme heat waves: 13.35 [13.22–13.48] days versus 7.70 [7.62–7.78] days, females: 13.72 [13.58–13.86] days versus 8.24 [8.18–8.31] days) (Figure 5C,D). Male prey size at maturity was influenced by both parental and offspring heat wave conditions, as well as by the interaction between the two factors, indicating TGE, WGP and TGP effects (Table 3). The body size of males originating from parents reared under extreme heat wave conditions was insensitive to offspring conditions (EM versus EE: *t*_134_ *=* 0.961; *p* = 0.772). WGP effects manifested in smaller body sizes under extreme heat wave conditions, when the males originated from parents reared under mild heat wave conditions (MM versus ME: *t*_134_ *=* 3.980; *p* = 0.0008). The TGP effects resulted in larger body sizes under mild heat wave conditions for males originated from parents reared under mild heat wave conditions (MM versus EM: *t*_134_ *=* 2.714; *p* = 0.036). These combined TGE, WGP and TGP effects led to similar male body sizes under extreme heat wave conditions (ME versus EE: *t*_134_ *=* 0.086; *p* = 1.000) (Figure 5E). Female body size was affected by parental heat wave conditions and the interaction between the main factors (Table 3). Female body sizes were insensitive to offspring heat wave conditions, when the females originated from parents reared under extreme heat waves (EM versus EE: *t*_162_ *=* 1.773; *p* = 0.310). Pooled over offspring heat wave conditions, females were larger when originating from parents reared under extreme heat waves, indicating non-plastic TGE (1042.08 [1024.27–1059.89] μm versus 1009.47 [999.22–1019.72] μm). TGP effects resulted in larger body sizes under mild heat waves, when the females originated from parents reared under extreme heat waves (EM versus MM: *t*_162_ *=* 4.018; *p* = 0.0008). These combined non-plastic TGE and TGP effects resulted in similar body sizes of the females under extreme heat waves (EE versus ME: *t*_162_ *=* 0.710; *p* = 0.893) (Figure 5F).

### 3.4. Species Comparisons: Heat Wave Effects on Predator-Prey Body Size Ratios and Developmental Time

**Figure 6:**
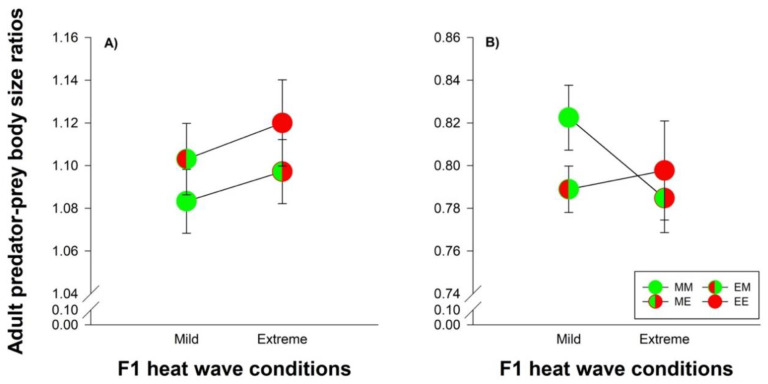
Adult male (**A**) and female (**B**) predator-prey body size ratios in dependence of their origin and exposure to heat waves. The circles illustrate the different heat wave combinations. Each circle was divided into two halves: the left half marks whether the parents (F0) were exposed to mild (green) or extreme (red) heat wave conditions, while the right half marks whether the offspring (F1) were exposed to mild (green) or extreme (red) heat wave conditions. Offspring, originating from parents reared under mild (M) or extreme (E) heat waves, were exposed to mild (M) or extreme (E) heat waves are labeled by the first and second upper case letters, respectively (MM = 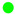, EM = 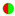, ME = 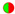 and EE = 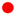). The lines connecting two circles refer to offspring experiencing the same heat wave condition as their parents. Vertical lines show the 95% confidence limits.

Male predators were larger than male prey, whereas female predators were smaller than female prey (body size ratios: 1.10 vs. 0.80). Male body size ratios were not affected by parental and offspring heat wave conditions (Figure 6A). Female body size became smaller under extreme heat waves, when the mites originated from parents reared under mild heat waves (MM versus ME: *t*_165_ = 3.581; *p* = 0.0005). The parental thermal environment of the female mites did not affect female body size ratios under extreme heat wave conditions (ME versus EE: *t*_180_ = 0.682; *p* = 0.496) (Figure 6B). 

**Figure 7:**
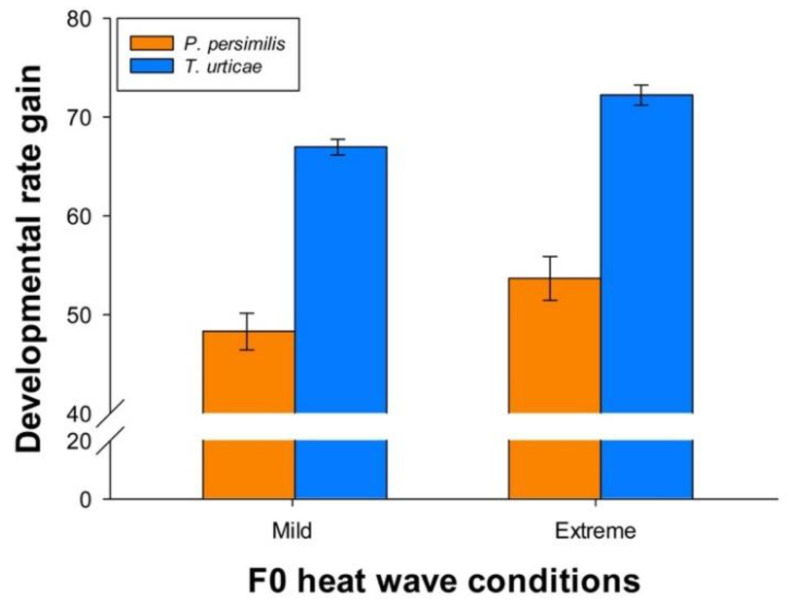
Percentage gains in developmental rates from mild to extreme heat waves during juvenile development of *P. persimilis* (orange bars) and *T. urticae* (blue bars), when their parents were exposed to mild or extreme heat waves. Vertical lines show the 95% confidence limits.

Irrespective of parental treatment, the prey developed relatively faster than the predator when heat wave conditions changed from mild to extreme. Thus, the predator increased its developmental rate with 51.10 % under extreme heat wave conditions compared with mild conditions, while the prey achieved an even greater advantage of higher temperatures, namely 69.52 % (Figure 7). Parental effects were reflected by the fact that predator offspring originating from parents exposed to extreme heat waves increased developmental rates by 53.66 [51.45–55.88]%, whereas the increase was 48.28 [46.42–50.15]% when their parents had been exposed to mild heat waves. The corresponding values for the prey were 72.23 [71.21–73.25]% and 66.97 [66.18–67.76]%, respectively.

## 4. Discussion

Our results revealed that both the parental and offspring thermal environment affected the life history traits of the predator *P. persimilis* and its prey *T. urticae* via WGP, TGP, and non-plastic TGE. First, only *T. urticae* parents, experienced to extreme heat waves, invested in larger egg sizes. Second, exclusively WGP resulted in faster development of both species and in higher consumption rates of predator and prey males and predator females under extreme heat wave conditions. Third, non-plastic TGE and WGP led to smaller body sizes of predator females under extreme heat waves. Fourth, non-plastic TGE and TGP resulted in equal body sizes of prey females under extreme heat wave conditions. Sixth, WGP and TGP were responsible for higher consumption rates of prey females under extreme heat waves. The same effects led to larger body sizes of prey males under mild heat waves, but to equal body sizes under extreme heat waves. Finally, the body sizes of predator males were insensitive to both parental- and offspring thermal environment.

### 4.1. Thermal Developmental Plasticity on Age and Size at Maturity: Adaptive or Not?

Following a general trend [46,47], both predator and prey exhibited higher consumption rates per capita under extreme heat waves. This extra energy was obviously not only invested in the maintenance of vital body functions under heat stress, but also in development, because both mite species reached earlier adulthood under extreme heat waves. Speeding up juvenile development should have two essential benefits for the mites. First, it reduces the exposure time of the heat-sensitive early developmental stages, which should lower juvenile mortality. Second, species with high capacities for population increase should profit more from increased developmental rates than from a proportionally equivalent increase of reproduction rates in relation to population growth [48,49]. These requirements apply for both the predator and its prey so that higher developmental rates should also result in higher intrinsic rates of increase under extreme heat waves. Body size at maturity of predator females followed the temperature-size (TS) rule (i.e., faster juvenile development, but smaller body size under high temperatures [50]). However, the rule did not apply to predator males and to prey males and females originating from parents that had experienced extreme heat waves. Their body sizes were insensitive to offspring heat wave conditions despite higher developmental rates. Such deviations from the TS rule (insensitive- or larger body size at higher temperatures) are documented both for terrestrial [4,51,52,53,54,55,56] and aquatic ectotherms [57,58]. Nonetheless, changes in age and size at maturity correspond to the TS rule under high temperatures for the majority of ectothermic species [50,59]. This fact raises the question whether small body size is an adaptive response to shifted selection pressures via higher temperatures [60,61]. Optimality models predict among others that an individual should develop fast at smaller sizes under high temperatures, when (a) higher temperatures are linked with higher juvenile mortality, and (b) large body size requires delayed maturation and lead to slower population growth [59]. Our juvenile mortality data of predator and prey, however, were very low (0.06 to 0.15) and not affected by heat waves. Additionally, insensitive body sizes under extreme heat waves were not coupled with prolonged juvenile development of predator and prey males and prey females. Along the same line, evidence for the selection of small body sizes via warmer environments is weak or missing both in endotherms and ectotherms [61,62,63,64], which put the adaptive value of small body sizes under high temperatures in question. Contrary, high temperatures are often associated with desiccation stress and small individuals are more prone to water loss because of their large surface area in relation to volume. Therefore, small body size should result in high costs via lowered heat- and desiccation resistance, as documented in ants [65,66], dung beetles [67], dung flies [68], and isopods [69].

Summing up, we argue that both fast development and insensitive body sizes are beneficial for the predator males and prey males and females, because it should lower juvenile mortality by reducing the time slot of heat-sensitive early developmental stages [10] and increase heat- and/or desiccation resistance under extreme heat waves [65,68]. Nonetheless, these irreversible responses to heat waves may incur costs later in life (e.g., adult survival, fecundity). The predator females, however, may suffer from heat stress under extreme heat waves and may overwhelm less prey during their juvenile development because of their reduced body sizes. 

### 4.2. Species-and Sex-Specific Responses to Heat Waves

First, irrespective of sex and parental thermal environment, both species exhibited higher food intake under extreme heat waves. Our findings indicate that this extra energy was directly involved in age-, but not size at maturity. Only prey females originating from parents that have experienced extreme heat waves gained an extra benefit: they had higher food intake rates compared to prey females originating from parents reared under mild heat waves, but they gained no benefit in relation to age and size at maturity. Other non-evaluated traits, important in the adult phase of prey females such as anti-predator behavior, adult longevity and number and size of eggs might be also positively affected by the higher food intake during juvenile development. Second, a potential cause of being smaller might be that growth rates decrease with progressive juvenile age at higher temperatures [70], which may explain the smaller female body sizes of the predator under extreme heat wave conditions. Contrary, body sizes of prey and predator males and prey females, originating from parents reared under extreme heat waves, were insensitive to offspring heat wave conditions, but the proximate causes for these findings could be species-specific. Only prey mothers manipulated egg sizes by the deposition of larger eggs, following the opposite trend in arthropods [71]. Indeed, predator mothers did not invest in large eggs, but they might increase the male egg quality by a transfer of nutrients to eggs. Both large egg sizes and the transfer of nutrients may contribute to unchanged body size. Third, the gain in the developmental rates due to extreme heat waves was much higher in the prey. Furthermore, shifts in female body size under extreme heat waves resulted in lower female predator-prey body size ratios, following a similar trend observed in the parental generation [10]. At first glance, these shifts in age and size at maturity may lower the predator effects per capita on prey densities under extreme heat waves, because (a) fast juvenile development shortening the time slot for predation on juvenile prey and (b) the shift in the body size ratios in favor of female prey reduces successful attacks of female predators. Lowering the interaction strength between predators and prey could decrease the predator-prey ratio and increase the time until the predators gain control of the prey [72], thereby jeopardizing biological control of spider mites by means of *P. persimilis*. However, prey was only exposed to heat stress, but not to predation risk, in our experiments. The mere presence of the predator can alter the life history traits of prey [35]. Hence, the strength and direction of shifts in age and size at maturity could be different from our results when prey is exposed both to heat waves and predation risk, making further experiments necessary.

Plastic responses allow for a fast adjustment to changed conditions and, as a consequence, heterogeneous environments should favor high phenotypic plasticity [14]. This was the case in relation to age at maturity, food consumption (predator and prey females and males) and size at maturity (predator females and prey males and females), when exposed to heat waves during juvenile development. Contrary, the body size of the predator males was almost unaffected by parental and offspring heat wave conditions. Interestingly, body size of the male predator was also insensitive to another environmental stressor: food shortage [31]. Male body size should be robust against environmental fluctuations when deviations from optimal size are linked with high fitness costs [73,74,75]. Except for size dimorphism and lower food intake of smaller males, both sexes have identical ecological niche demands. Thus, it is more likely that male body size canalization was the result of sexual selection rather than natural selection. For example, being smaller than standard-sized males incur high fitness costs, because males are frequently engaged in male-male combats for receptive females [76] and small *P. persimilis* males are inferior in mate competition [77]. Additionally, small predator males produce fewer spermatophores and have a lower lifetime reproductive success than standard-sized males [78,79]. Based on these findings, it seems reasonable that smaller male phenotypes are not favored by sexual selection. On the other hand, which mechanisms preclude the male mites from becoming larger? An explanation might be that males have to overcome the gravity when climbing in the vertical plant structures to search for receptive females among leaves within plants. According to the gravity hypothesis, climbing speed should be lower for larger males [80]. As a consequence, larger males should suffer from lower mate encounter rates and mating success. However, empirical evidence for supporting the gravity hypothesis is low [81,82]. More likely, inherent high costs of plasticity and/or limited genetic variation may constrain the evolution of larger male body sizes in *P. persimilis* [14].

### 4.3. Potential Experimental Shortcomings

Research on TGP effects has gained increased attention in the last decade in the scientific community. However, reviews also highlighted potential methodological deficits [11,83,84], which may lead to the over- or underestimation of TGP effects. First, parents can only adjust offspring phenotypes correctly to their environment if the parental environment is a reliable predictor of the offspring environment [85]. Both predator and prey are characterized by rapid juvenile development, reaching adulthood within few days, thereby making the exposure to heat waves of both parents and their offspring likely, at least partly. Second, a clear separation of plastic from genetic adaptations to heat stress can be easily managed for parthenogenetic species such as water fleas or aphids [86,87]. However, our offspring generations developed from sexually reproducing parents. A potential alternative would be the use of isofemale lines. This approach was not conducted in our experiments, because it drastically boosts the temporal expenditure of the experiments. Nonetheless, we conclude that the trait shifts were mainly attributed to plastic responses to heat stress, because genetic adaptations become dominant after several, but not two generations as in our experiments. Moreover, offspring survival was high and not affected by heat waves, which excludes selective mortality effects [83]. Third, the separation of parental and offspring environment in the early offspring development is a challenge. Here, the mean egg age (±3 h) correlates with 7% (for the predator) and 3.5% (for prey) of the egg development, which should limit confounding thermal effects of the parental environment [83]. But an explicit verification of TGP effects implies that such modifications are also apparent in the F2 generation, because the primordial offspring germ cells may experience the parental environment before egg deposition [11]. Under practical aspects, however, grandparental environment should hardly correspond with offspring heat wave conditions because of the short periods of heat waves relative to the consecutive development of three mite generations despite of ongoing climate warming. Thus, grandparental trans-generational modifications are likely maladaptive for juvenile offspring under natural heat wave conditions. 

## 5. Conclusions

Our study showed the ability of predator and prey offspring to react to heat stress via within-generational and trans-generational developmental plasticity, which may allow them to keep pace with fast changing thermal environments like heat waves. This ability will be of major importance for predator-prey interactions, as heat waves should increase in frequency, duration and intensity in the next decades [2]. Nonetheless, other interesting aspects of TGP were not scrutinized in our experiments. Are only mothers responsible for the modifications of offspring traits or do fathers also contribute to TGP [11]? Is heat wave exposure during short-term sensitive phases in parental development (e.g., early- or late juvenile development) sufficient to trigger similar TGP effects [11]? Are the trans-generational effects induced by epigenetic variation (e.g., DNA-methylation and chromatin structure) or other mechanisms (e.g., transmission of nutrients and hormones) [11]? Do heat waves activate the expression of heat shock proteins in predator and/or prey offspring? Such investigations should provide a deeper insight in the mechanisms of TGP in ectothermic species.

Our findings of heat wave effects on parental [10] and offspring development (results presented here) may denote the inferiority of the predator to cope with heat waves compared to its preferred prey. However, the potential implications for spider mite control in crop systems are limited because our experimental design did not incorporate other ecological niche dimensions, which strongly affect the outcome of predator-prey interactions in more complex natural settings. Further investigations are needed to understand how the mere presence of the predator may influence development, reproduction and migration in prey [35,88], both at the individual and population level, to verify the suppression potential of *P. persimilis* against *T. urticae*, under heat waves.

## Figures and Tables

**Table 1:** Heat wave effects (mild or extreme heat waves) on juvenile survival and offspring sex ratio rates of offspring (F1) originating from parents (F0) reared under either mild or extreme heat wave conditions. Data were analyzed by means of logistic regression (PROC LOGISTIC in SAS). The Hosmer-Lemeshow test was used to assess the goodness-of-fit of the logistic model using F0 and F1 as predictor variables. Large *p*-values signify that the agreement between observed and predicted values was good.

Factor	Source of Variation	*Phytoseiulus persimilis*			*Tetranychus urticae*		
		Wald’s χ^2^	df	*p*	Wald’s χ^2^	df	*p*
Survival	F0	2.076	1	0.1498	0.436	1	0.5090
	F1	2.770	1	0.0960	2.945	1	0.0862
	F0*F1	0.316	1	0.5742	0.253	1	0.6149
	Goodness-of fit	0.318	2	0.8532	0.254	2	0.8808
Sex ratio	F0	0.034	1	0.8531	0.028	1	0.8663
	F1	2.272	1	0.1317	0.028	1	0.8663
	F0*F1	0.012	1	0.9143	2.217	1	0.1365
	Goodness-of-fit	0.012	2	0.9942	2.223	2	0.3291

**Table 2:** Heat wave effects (mild or extreme heat waves) on age and size at maturity, and food consumption of offspring (F1) deriving from *P. persimilis* parents experiencing either mild or extreme heat wave conditions (F0). *p*-values in bold indicate a significant effect (*p <* 0.05). Data were analyzed by a mixed ANOVA (PROC GLM in SAS) using F0 as a random factor and F1 as a fixed factor. The parameters associated with F0 (*β*_1_), F1 (*β*_2_), and F0*F1 (*β*_3_) indicate the relative contribution of non-plastic trans-generational effects (TGE), within-generational plastic effects (WGP), and trans-generational plastic effects, respectively, to the overall treatment response. A positive parameter value implies that extreme heat waves is expected to increase the trait in focus, while a negative value is expected to decrease the response.

Factor	Source of Variation	*P. persimilis Males*				*P. persimilis Females*			
		$\hat{\beta }$	*t*	df	*p*	$\hat{\beta }$	*t*	df	*p*
**Age at maturity**	F0	−0.1848	−1.34	109	0.1816	−0.0040	−0.05	176	0.9638
	F1	−2.1126	−13.68	109	**<0.0001**	−2.0436	−22.86	176	**<0.0001**
	F0*F1	−0.0447	−0.21	109	0.8317	−0.1543	−1.26	176	0.2085
**Size at maturity**	F0	−6.8912	−1.44	109	0.1525	9.8281	2.21	176	**0.0281**
	F1	−9.2020	−1.71	109	0.0896	−13.1326	−2.92	176	**0.0040**
	F0*F1	16.1598	2.21	109	**0.0290**	6.8832	1.12	176	0.2653
**Food consumption**	F0	0.8843	1.99	109	**0.0489**	0.0604	0.19	176	0.8503
	F1	3.9666	7.95	109	**<0.0001**	3.8859	11.98	176	**<0.0001**
	F0*F1	−1.3177	−1.94	109	0.0544	−0.3847	−0.87	176	0.3869

**Table 3:** Heat wave effects (mild or extreme heat waves) on age and size at maturity, and the food consumption of offspring (F1) deriving from *T. urticae* parents experiencing either mild or extreme heat wave conditions (F0). *p*-values in bold indicate a significant effect (*p <* 0.05). Data were analyzed by a mixed ANOVA (PROC GLM in SAS) using F0 as a random factor and F1 as a fixed factor. The parameters associated with F0 (*β*_1_), F1 (*β*_2_), and F0*F1 (*β*_3_) indicate the relative contribution of non-plastic trans-generational effects (TGE), within-generational plastic effects (WGP), and trans-generational plastic effects, respectively, to the overall treatment response. A positive parameter value implies that extreme heat waves is expected to increase the trait in focus, while a negative value is expected to decrease the response.

Factor	Source of Variation	*T. urticae Males*				*T. urticae Females*			
		$\hat{\beta }$	*t*	df	*p*	$\hat{\beta }$	*t*	df	*p*
**Age at maturity**	F0	0.1310	1.19	134	0.2366	0.2464	2.30	162	**0.0225**
	F1	−5.5048	−53.05	134	**<0.0001**	−5.3386	−52.40	162	**<0.0001**
	F0*F1	−0.2929	−1.95	134	0.0529	−0.2569	−1.77	162	0.0788
**Size at maturity**	F0	−14.4298	−2.72	134	**0.0073**	60.3511	4.02	162	**<0.0001**
	F1	−19.8578	−3.98	134	**<0.0001**	25.6546	1.79	162	0.0747
	F0*F1	14.8522	2.06	134	**0.0414**	−50.6480	−2.49	162	**0.0137**
**Food consumption**	F0	−0.0708	−0.68	134	0.4954	0.1062	0.87	162	0.3861
	F1	0.4773	4.89	134	**<0.0001**	1.0373	8.92	162	**<0.0001**
	F0*F1	0.1983	1.41	134	0.1620	0.6146	3.72	162	**0.0003**

## Data Availability

The data that support the findings of this study are openly available in Zenodo at https://doi.org/10.5281/zenodo.6637891.

## Supplementary Materials

The following supporting information can be downloaded at: https://www.mdpi.com/article/10.3390/biology11081123/s1, Figure S1: Diurnal variations in temperature (blue lines) and humidity (pink lines) mimicking ex-treme (A) and mild (B) heat waves at long-day conditions (L:D = 16 h:8 h).
