# Supplementary material for: Trans- and Within-Generational Developmental Plasticity May Benefit the Prey but Not Its Predator during Heat Waves
Source: Biology (Basel). 2022 Jul 27;11(8):1123. doi: 10.3390/biology11081123 (PMC9404866; doi:10.3390/biology11081123)
Supplement: Supplementary file 1 [file biology-11-01123-s001.zip › biology-1792468-supplementary.pdf]

Supplementary material

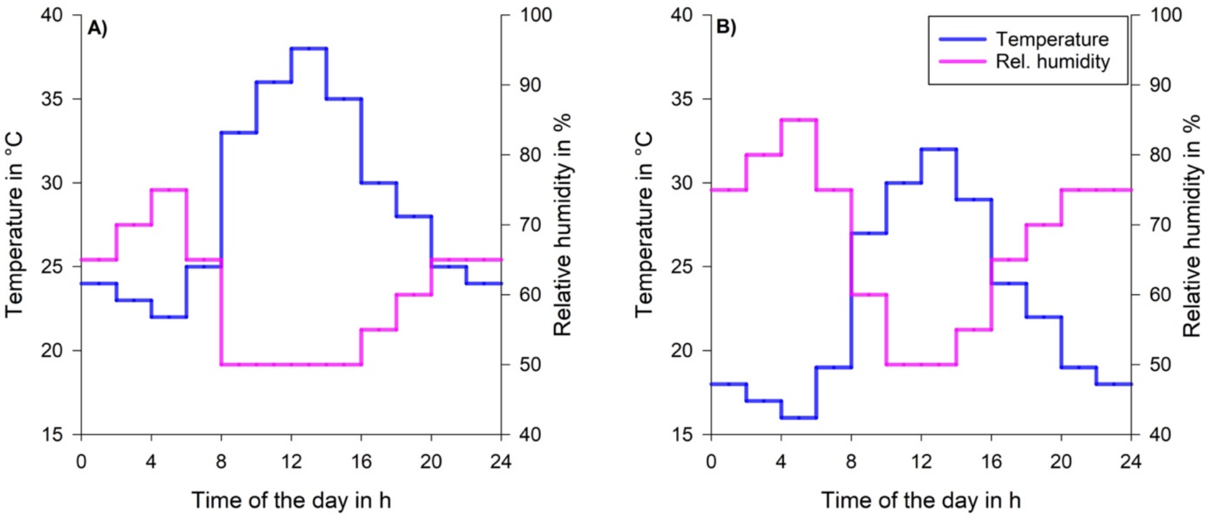

Figure S1. Diurnal variations in temperature (blue lines) and humidity (pink lines) mimicking extreme (A) and mild (B) heat waves at long-day conditions (L:D = 16h:8h) (mild heat waves: T_{mean} = 22.6°C, T_{max} = 32.0°C, T_{min} = 16°C, RH_{mean} = 67.9%, RH_{max} = 85.0%, RH_{min} = 50.0%; extreme heat waves: T_{mean} = 28.6°C; T_{max} = 38.0°C, T_{min} = 22.0°C; RH_{mean} = 60.0%, RH_{max} = 75.0%, RH_{min} = 50.0%).
